# Intra-uterine Growth Restriction Downregulates the Hepatic Toll Like Receptor-4 Expression and Function

**DOI:** 10.1080/17402520400008905

**Published:** 2005-03

**Authors:** Ozlem Equils, Sapna Singh, Semra Karaburun, Daning Lu, Manikkavasagar Thamotharan, Sherin U. Devaskar

**Affiliations:** 1 Department of Pediatrics Steven Spielberg Pediatric Research Center Burns and Allen Research Institute Cedars-Sinai Medical Center David Geffen School of Medicine at UCLA Los Angeles CA 90048 USA; 2 Pennsylvania State University Jefferson Medical College Philadelphia 19107 USA; 3 Department of Pediatrics David Geffen School of Medicine at UCLA and Mattel Children's Hospital at UCLA Los Angeles CA 90024 USA

## Abstract

Maternal starvation is a significant cause of intrauterine growth restriction (IUGR) in the world and increases the risk of infection in the neonate. We examined the effect of maternal starvation on Toll like receptor (TLR)4 expression in hepatic, splenic and intestinal tissues obtained from the adult IUGR offspring of prenatal calorie restricted rats. The hepatic TLR4 protein concentration was undetectable in the IUGR rats that had restricted milk intake during the suckling period (SM/SP; *n* = 4, *p* < 0.05) as compared to the normal growth controls (CM/CP; *n*=4), and access to ad lib milk intake during the sucking period partially corrected the hepatic TLR4 expression (SM/CP; *n* = 4). IUGR had no effect on the splenic (*n* = 4) or intestinal (*n* = 4) TLR4 mRNA levels. In the liver, IUGR led to a 20% increase in baseline tumor necrosis factor (TNF)-α mRNA expression ( *p* < 0.03) and a 70% increase in interleukin-1β (IL-1β) mRNA expression ( *p* < 0.008) as compared to the control rats (CM/CP; *n* = 7). LPS-induced hepatic TNF-α release was significantly higher in SM/SP as compared to CM/CP. We propose that IUGR dysregulates TLR4 expression and function in the offspring, which may help explain the increased risk of Gram-negative sepsis and inflammatory diseases in this population.


*Clinical & Developmental Immunology*, March 2005; 12(1): 59–66

Taylor & Francis

Taylor & Francis Group

### Intra-uterine growth restriction downregulates the hepatic toll like receptor-4 expression and function

OZLEM EQUILS[1], SAPNA SINGH[2], SEMRA KARABURUN[1], DANING LU[1], MANIKKAVASAGAR THAMOTHARAN[3], & SHERIN U. DEVASKAR[3]

[1]*Department of Pediatrics, Steven Spielberg Pediatric Research Center, Burns and Allen Research Institute, Cedars-Sinai Medical Center David Geffen School of Medicine at UCLA, Los Angeles, CA 90048, USA,* [2]*Pennsylvania State University Jefferson Medical College, Philadelphia 19107, USA, and* [3]*Department of Pediatrics, David Geffen School of Medicine at UCLA and Mattel Children's Hospital at UCLA, Los Angeles, CA 90024, USA*

**Abstract**
Maternal starvation is a significant cause of intrauterine growth restriction (IUGR) in the world and increases the risk of infection in the neonate. We examined the effect of maternal starvation on Toll like receptor (TLR)4 expression in hepatic, splenic and intestinal tissues obtained from the adult IUGR offspring of prenatal calorie restricted rats. The hepatic TLR4 protein concentration was undetectable in the IUGR rats that had restricted milk intake during the suckling period (SM/SP; $n = 4$, $p < 0.05$) as compared to the normal growth controls (CM/CP; $n = 4$), and access to *ad lib* milk intake during the sucking period partially corrected the hepatic TLR4 expression (SM/CP; $n = 4$). IUGR had no effect on the splenic ($n = 4$) or intestinal ($n = 4$) TLR4 mRNA levels. In the liver, IUGR led to a 20% increase in baseline tumor necrosis factor (TNF)-α mRNA expression ($p < 0.03$) and a 70% increase in interleukin-1β (IL-1β) mRNA expression ($p < 0.008$) as compared to the control rats (CM/CP; $n = 7$). LPS-induced hepatic TNF-α release was significantly higher in SM/SP as compared to CM/CP. We propose that IUGR dysregulates TLR4 expression and function in the offspring, which may help explain the increased risk of Gram-negative sepsis and inflammatory diseases in this population.

**Keywords:** *Diabetes mellitus, intrauterine growth retardation, neonate, sepsis, starvation, toll like receptor*

**Abbreviations:** *LPS, lipopolysaccharide; TLR, Toll-like receptor; TNF, tumor necrosis factor; IL, interleukin; IUGR, intrauterine growth restriction*

### Introduction

In addition to genetic factors, environmental influences such as maternal nutrition play an important role in influencing the developing human immune system (reviewed in Chandra (2002)). Adverse factors that impair fetal growth such as maternal malnutrition, hinder immunological maturation as well (Moscatelli et al. 1976, Chandra 1975a,b 1981, Ferguson 1978, Hasselbalch et al. 1999). Both animal and human data suggest that those who are small for gestation show persistent immunological impairment for several months or even years (Chandra 1975c, Beach et al. 1982), and that they are at increased risk of infections with Gram-negative bacteria during the neonatal period and childhood (Simchen et al. 2000).

Toll-like receptors (TLRs) have recently been identified to mediate the innate immune responses to microbial antigens (Modlin 2002a), and TLR4 is the receptor for enteric Gram-negative bacterial cell wall component lipopolysaccharide (LPS) (reviewed in Beutler et al. (2001)). The interaction between TLR4 and LPS results in the secretion of antibacterial peptides, defensins and proinflammatory cytokines such as tumor necrosis factor (TNF)-α and interleukin (IL)-6, which initiate an inflammatory response to clear the invading organism. Furthermore,

Correspondence: O. Equils, Address: Department of Pediatrics, Cedars-Sinai Medical Center, 8700 Beverly Blvd, Room 4220, Los Angeles, CA, USA 90048. Tel: 1 310 423 4471. Fax: 1 310 423 8284. E-mail: ozlem.equils@cshs.org

ISSN 1740-2522 print/ISSN 1740-2530 online © 2005 Taylor & Francis Group Ltd.
DOI: 10.1080/17402520400008905

the inflammatory response results in the recruitment of cells of adaptive immunity to initiate clearance of the pathogens by generating a specific immune response. Hence, defects at the level of expression of TLR4 could contribute to poor recruitment of antigen presenting cells, and T and B cells at the site of inflammation, resulting in suboptimal adaptive immune responses leading to increased risk of infections with Gram-negative bacteria.

Currently, the data on the effect of maternal malnutrition on immune system development is limited to the deficits in the development of adaptive immunity and there are no data on whether IUGR influences the expression of innate immune system receptors, TLRs. To address this, we examined the expression of TLR4 in liver, spleen and intestinal tissue obtained from adult IUGR offspring of intrauterine starved rats by semi-quantitative reverse transcriptase (RT)-PCR and Western blot analyses. We showed that hepatic TLR4 protein expression decreased significantly in both the adult IUGR offspring of rats that had restricted and *ad lib* access to milk intake during the suckling period. The baseline hepatic IL-1β and TNF-α mRNA expression, which have previously been shown to regulate TLR4 expression (Alves-Rosa et al. 2002) and play a role in the development of insulin resistant phenotype (Pickup and Crook 1998) were higher in the IUGR offspring. Despite lower levels of TLR4 expression, LPS-induced TNF-α release was higher in the IUGR as compared to the control rats.

### Materials and methods

### *Animals*

Sprague–Dawley rats (250–300 g) were housed in individual cages, exposed to 12 h light/dark cycles at 21–23°C, and allowed access to standard rat chow (Purina Co., St. Louis, Mo.) *ad libitum* as approved by the Animal Research Committee of the University of California, Los Angeles as per the guidelines of the National Institutes of Health.

*Maternal starvation model*. We generated the adult offspring of intrauterine growth restricted (IUGR) rats as described previously (Holemans et al. 1997). Briefly, pregnant rats received 50% of their daily food intake beginning from day 11–21 of gestation as compared to their control counterparts who received *ad lib* access to rat chow. This led to caloric restriction during late gestation.

*Postnatal animal maintenance*. At birth, the litter size was culled to six. At day 1, rats born to starved mothers were either reared by mothers continued to be food restricted, receiving only 11 g/day throughout lactation or a control mother with *ad lib* access to rat chow. The control pups were also either reared by their own mother or fostered to a mother receiving restricted food through out lactation. Thus four groups were created, with control mothers rearing control (CM/CP) or starved (CM/SP) pups and the starved mothers rearing starved (SM/SP) or control (SM/CP) pups throughout the suckling phase. At d21, the pups were weaned from the mother and maintained in individual cages until d180 of life.

*Tissue collection*. Liver, spleen and intestine were obtained from the anesthetized animal which continued to maintain good organ blood flow, tissues were immediately snap frozen in liquid nitrogen, and stored at −70°C until analyses.

### *Cytokine and TLR4 mRNA expression*

About 30–50 mg of frozen rat tissue was lysed. Total RNA was isolated using Trizol (Invitrogen, Carlsbad, CA) according to the manufacturer's instructions. For reverse transcription 1 μg of RNA and 1 μl of Oligo dT primer (0.5 μg, Invitrogen, Carlsbad, CA) were added to DEPC'd $H_2O$ for final reaction volume of 10 μl, mixed and incubated at 65°C for 10 min, and then at room temperature for 2 min. Ten microliters of Reverse Transcription Buffer (4 μl of 5 × First Strand Buffer, 2 μl of 0.1 M DTT, 1 μl of 2.5 mM dNTPs, 1 μl of RNase Inhibitor, 1 μl of M-MLV (Invitrogen, Carlsbad California) and 1 μl of DEPC'd $H_2O$) was added. Twenty microliters of this mixture in a tube was placed in a water bath at 37°C for 90 min, and then transferred to a water bath maintained at a temperature of 65°C for 15 min. The cDNA was stored at −20°C for PCR and future studies. Semi-quantitative PCR reaction volume was 25 μl. Twenty microliters of master buffer contained 2.5 μl of 10 × PCR Reaction Buffer (3 mM MgCl2), 2.5 μl of 2 mM dNTPs, 2 μl of 0.5 pm rat GAPDH primers (GAPDH sense, 5′-ATC AAC GAC CCC TTC ATT GA-3′; GAPDH antisense, 5′-AGA TCC ACA ACG GAT ACA TT-3′ (Genebank accession #NM017008)), 2 μl of test primers, 0.25 μl of Taq DNApolymerase (BRL) and 10.75 μl of water. Other primers used were: rat TNF-α primers (rat TNF-α sense, 5′-AAG TCA GCC TCC TCT CTG CC-3′; TNF-α antisense, 5′-AAG TAG ACC TGC CCG GAC TC-3′ (Genebank Accession #MN012675)); rat IL-1β primers (rat IL-1β sense, 5′-CAT CTT TGA AGA AGA GCC CG-3′ and IL-1β antisense, 5′-AGC TTT CAG CTC ACA TGG GT-3′ (Genebank Accession #NM031512)). Five microliters of cDNA was added to 20 μl of PCR buffer, mixed and briefly centrifuged, PCR conditions were: prewarming at 95°C for 5 min; an annealing temperature of 60°C

for 30″, 72°C for 2′; 30 cycles and extension at 72°C for 5 min. The PCR products were electrophoresed on 4% a NuSieve agarose gel (Reliant Gel System, BMA) at 50 V for 2 h. The image was photographed using a KODAK DC290 Digital Camera, the net intensity of the PCR bands was analyzed on an AlphaImager 2000 densitometer (Alpha Innotech Corp.). AlphaEase software (Alpha Innotech Corp.) was used to compare the density of products and correct for intensity of GAPDH. Both the test cDNA and the GAPDH cDNA were amplified in the same reaction tube.

*Preparation of protein extracts*

Liver, spleen and intestinal tissues were homogenized in buffer containing (2% nonidet P-40, 300 mM NaCl, 50 mM HEPES (pH 7.9), 2 mM EDTA, 4% glycerol, 0.1 mM dithiothreitol (DTT), 1.0 μg of leupeptin per ml, sodium orthovanadate (Na3VO4) (1 mM pH = 12.5–13.5), 5.0 μg of trypsin per ml, 2.0 μg of aprotinin per ml, phenylmethylsulphonyl fluoride (PMSF) (1 mN pH = 7.0–7.5) and protease inhibitor cocktail (Sigma). After transfer to a 1.5-ml tube, the debris was pelleted by centrifugation at 1500*g* for 20 min. Supernatants containing proteins were aliquoted and stored at 70°C. The protein concentration was determined by the Bradford assay.

*Western blot analysis*

The cell lysates were resolved by SDS-PAGE and transferred on to a PVDF membrane (Bio-Rad Laboratories, Hercules, CA). The membrane was blotted with Anti-TLR4 rabbit polyclonal primary antibody (Santa Cruz Biotechnology Inc., Santa Cruz, CA sc-10741) and the secondary antibody, which is donkey anti-rabbit IgG conjugated to horseradish peroxidase (HRP) (Amersham Biosciences, Piscataway, NJ), and visualized with an enhanced chemiluminescence system (Amersham Chemiluminescence HRP, Piscataway, NJ).

*Generation of ex vivo hepatocytes*

Liver tissue from rats was used to generate primary cell lines. Briefly, 0.5 g of the tissue was placed in Dissection Medium that contains HBSS and centrifuged at 700 rpm for 3 min twice. Tissue pieces were then transferred to a large petridish and minced into small pieces using sterile scissors. All tissue pieces were then transferred to a 50 ml centrifuge tube and washed at 700 rpm for 3 min. Supernatant was discarded and trypsin-EDTA mixture was added to the tube, which was incubated at 37°C for 10 min and swirled briefly every minute. The cells were then washed twice with PBS at 700 rpm for 3 min; tissue pieces were transferred to a nylon bag and marshed with a pestle. The cell suspension was passed through mesh screen (first larger size mesh, then smaller size mesh). The cells were collected in a 50-ml tube, and washed twice at 700 rpm for 3 min. The cells were then split into two T150s' flasks and incubated at 37°C in minimum essential medium (MEM), with 20% horse serum and 10% FBS. At passage numbers 2–4, 50,000 cells/well were plated in quadruplicate in 12-well plates and stimulated with media or 100 ng/ml LPS/TLR4 agonist for 5 h, supernatant was harvested for cytokine analysis.

*TNF-α ELISA assay*

*Ex vivo* hepatocytes were stimulated with LPS for 5 h and the supernatant was harvested and frozen for batch TNF-α ELISA assay according to the manufacturers protocol (Becton Dickinson).

*Statistics*

Results were presented as mean ± SD, and differences were analyzed for statistical significance ($p > 0.05$) by the Student $t$ test. The experiments were repeated at least three times with similar results.

### Results

*Intrauterine growth restriction down-regulates hepatic TLR4 protein expression*

Both animal and human data suggest that IUGR increases the risk of sepsis and death from Gram-negative bacterial infections during the neonatal period (Simchen et al. 2000). Lipopolysaccharide (LPS) is the main component of Gram-negative bacterial cell wall and is a potent inducer of the innate immune responses (Wenzel et al. 1996, Rietschel et al. 1996) through TLR4 (Poltorak et al. 1998, Hoshino et al. 1999). We hypothesized that IUGR down-modulates the TLR4 expression and this may help explain the increased risk of Gram-negative infections. In order to test this hypothesis we used Western blot analyses and showed that hepatic TLR4 protein levels decreased significantly in both the adult IUGR offspring of prenatal calorie restricted rats that had restricted (SM/SP, $n = 4$) and *ad lib* milk intake (SM/CP, $n = 4$) during the suckling period as compared to the control rats (CM/CP) (**Figure** 1A). IUGR did not modulate the splenic or intestinal TLR4 mRNA levels (data not shown).

Next, we generated primary hepatocytes using liver tissues obtained from CM/CP and SM/SP, and examined TLR4 mRNA and protein expression in these cells. We observed that, similar to liver homogenates, hepatocyte TLR4 mRNA (data not shown) and protein levels were lower in SM/SP ($n = 3$) as compared to CM/CP ($n = 3$) (**Figure** 1B).

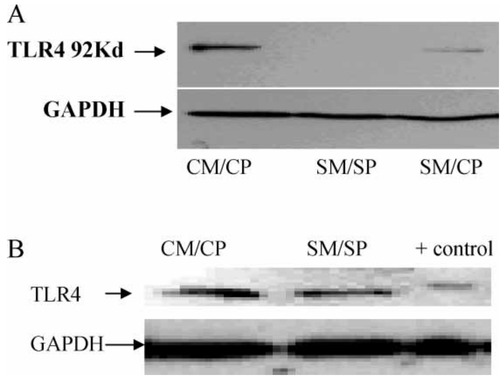

Figure 1. (A) The hepatic TLR4 protein expression is lower in the adult IUGR offspring of prenatal calorie restricted rats. We assessed the effect of IUGR on hepatic TLR4 protein expression by Western blot analysis, and observed that IUGR led to decreased hepatic TLR4 protein levels in the adult IUGR offspring of prenatal calorie restricted rats that had restricted milk intake during the sucking period (SM/SP; $n = 4$) as compared to control rats (CM/CP; $n = 4$), and that access to *ad lib* milk intake during the suckling period partially corrected the effect of IUGR on hepatic TLR4 protein expression. The experiment was repeated at least three times with similar results. (B) The hepatic TLR4 protein expression is lower in SM/SP as compared to CM/CP. Since hepatocytes were grown in media containing growth factors, we wanted to assess whether the expression level of TLR4 was altered in SM/SP. We generated primary hepatocyte cell lines as described under "Methods" and assessed TLR4 expression by Western blot analysis. Cell lysate from Santa Cruz (Cat #sc-2209 H-60) was used for TLR4 positive control. We observed that TLR4 protein level was significantly lower in the SM/SP ($n = 2$) as compared to CM/CP ($n = 2$). The data shown is the representative of at least three similar experiments.

These data suggest that *in-utero* nutrient restriction, which lead to IUGR, diminishes the hepatic TLR4 protein expression.

### *IUGR increases the baseline liver homogenate IL-1β and TNF-α mRNA expression*

Previous studies investigating the regulation of TLR gene expression have shown that exposure to pro-inflammatory cytokines (i.e. IL-1β and TNF-α) modulated the TLR gene expression and that the effect of cytokines on TLR expression varied between different cell types. The administration of IL-1 down-regulated the TLR4 expression in murine peritoneal macrophages (Alves-Rosa et al. 2002) and in human monocytic THP-1 cell line (Zarember and Godowski 2002), whereas it increased the TLR4 expression in primary human monocytes and peripheral blood mononuclear cells (Muzio et al. 2000) and had no effect on TLR4 mRNA levels in murine hepatocytes (Matsumura et al. 2000). Similarly, TNF-α exposure decreased the TLR4 expression in THP-1 cells (Zarember and Godowski 2002) but led to increased TLR4 expression in primary human monocytes and peripheral blood mononuclear cells (Muzio et al. 2000).

Since our data suggests that IUGR secondary to *in-utero* calorie restriction modulates the hepatic TLR4 levels we investigated whether the effect of IUGR on hepatic TLR4 expression was secondary to baseline hepatic IL-1β and TNF-α expression. Indeed, both IL-1β (Figure 2A) and TNF-α (Figure 2B) mRNA levels were significantly higher in the liver homogenates obtained from SM/SP and SM/CP as compared to CM/CP. These data suggest that the effect of IUGR on hepatic TLR4 expression may be due to the dysregulation of baseline cytokine expression in the liver and this observation may help explain the propensity of IUGR offsprings to later develop type 2 diabetes and atherosclerotic heart disease, which are inflammatory adult diseases.

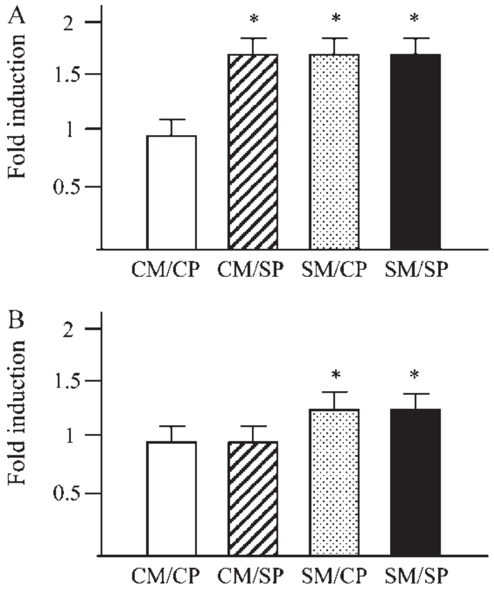

Figure 2. (A) The baseline IL-1β mRNA expression is increased in the hepatic homogenate obtained from adult IUGR offspring of prenatal calorie restricted rats. We assessed the baseline hepatic IL-1β mRNA levels by using semi-quantitative RTPCR. The results were normalized to GAPDH expression and are expressed as fold increase above the control (CM/CP). Hepatic IL-1β mRNA expressions were significantly higher in the SM/SP ($n = 7$) and SM/CP ($n = 6$) as compared to CM/CP ($n = 7$). Access to *ad lib* milk intake during suckling period (SM/CP) did not lead to a significant decrease in baseline IL-1β mRNA levels as compared to SM/SP. The hepatic IL-1β mRNA expressions in rats that were born to control mothers but starved during the suckling period (CM/SP; $n = 7$) were significantly higher than those observed in CM/CP. $*p \leq 0.05$. (B) The baseline hepatic TNF-α mRNA expression is increased in the adult IUGR offspring of prenatal calorie restricted rats. We assessed the baseline hepatic TNF-α mRNA levels by using semi-quantitative RT-PCR. The results were normalized to GAPDH expression and are expressed as fold increase above the control (CM/CP). Hepatic TNF-α mRNA expressions were significantly higher in the SM/SP ($n = 7$) and SM/CP ($n = 6$) as compared to CM/CP ($n = 7$) and CM/SP ($n = 7$). Access to *ad lib* milk intake during suckling period (SM/CP) did not lead to a significant decrease in baseline TNF-α mRNA levels as compared to SM/SP. The hepatic TNF-α mRNA expression in CM/SP was similar to that observed in CM/CP.

*LPS-induced TNF-α release is higher in the ex vivo hepatocytes obtained from the adult IUGR rats*

Recently, macrophages obtained from aged mice have been shown to express significantly lower levels of TLRs and secreted significantly lower levels of IL-6 and TNF-α when stimulated with TLR ligands as compared to the young mice (Renshaw et al. 2002). The authors concluded that the decreased TLR expression might lead to diminished TLR function and innate immune responses in the elderly, and help explain the increased risk of infections in this population.

We examined the effect of IUGR on TLR4 function by isolating *ex vivo* primary hepatocytes from SM/SP and CM/CP and stimulating them with either media or LPS for 5 h and measuring the supernatant for TNF-α release by an ELISA assay. We observed that LPS-induced TNF-α release was higher in the hepatocytes obtained from SM/SP ($n = 2$) as compared to the levels observed in CM/CP ($n = 2$) (**Figure** 3) despite lower TLR4 expression in SM/SP (Figure 3B). These results suggest that the innate immune responses are dysregulated in the IUGR. Sepsis is due to exaggerated immune activation upon antigenic stimulation; our results may help explain the increased risk of sepsis in the IUGR population. And in this population, the propensity to develop inflammatory conditions such as type 2 diabetes and atherosclerosis may be partly explained by the dysregulated immune activation.

### Discussion

Prenatal and early postnatal starvation have been linked to deficits in several aspects of adaptive immunity, to involution of lymphoid tissues such as the thymus, and to suppression of antibody responses to vaccination (Moscatelli et al. 1976, Chandra 1975a,b, Chandra 1981, Ferguson 1978, Moore 1998) as well as the development of atopy and autoimmune disease during adult life (Phillips et al. 1993, Godfrey et al. 1994). Here, we show that IUGR leads to an increase in the baseline hepatic IL-1β and TNF-α mRNA levels, diminishes the hepatic TLR4 mRNA and protein concentrations and augments the LPS-TNF-α responses in the adult offspring of intrauterine starved rats, and that access to *ad lib* milk intake during the suckling period partially corrects the IUGR effect on TLR4 protein expression; however, the baseline hepatic IL-1β and TNF-α mRNA levels are still elevated in the SM/CP. Interestingly, a recent study investigating the effect of prematurity on TLR4 expression showed high levels of hepatic TLR4 expression regardless of age (Harju et al. 2001).

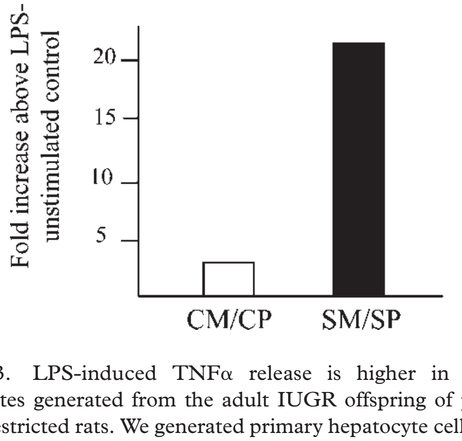

Figure 3. LPS-induced TNFα release is higher in *ex vivo* hepatocytes generated from the adult IUGR offspring of prenatal calorie restricted rats. We generated primary hepatocyte cell lines as described under "Methods" and assessed the LPS-induced TNF-α release by stimulating them for 5 h with purified LPS (100 ng/ml), and observed that the TNF-α release was significantly higher in the SM/SP ($n = 2$) as compared to CM/CP ($n = 2$). The data shown is the representative of at least three similar experiments.

Upon infection, TLRs recognize the components common to the pathogens that are not normally found in the host and play a key role in the initiation of early innate immune responses (reviewed in Modlin (2002b)). TLRs also mediate the initiation of adaptive immune responses through induction of co-stimulatory molecules and release of proinflammatory cytokines (reviewed in Barton and Medzhitov (2002)). Moreover, TLR signaling stimulates the maturation and migration of dendritic cells to the lymph nodes (Biragyn et al. 2002). Therefore diminished expression and function of TLRs may help explain the poor adaptive immune responses and increased risk of infections in the IUGR newborn.

TLR4 has recently been identified as the main receptor for enteric Gram-negative bacterial LPS together with CD14 and MD2 (Beutler 2002). TLR4 mediates the immune activation to a variety of agents including diterpene taxol, a widely used anticancer drug (Miyake et al. 2000), F protein of respiratory syncytial virus (RSV) (Kawasaki et al. 2000), chlamydial heat shock protein (cHSP) 60 (Kurt-Jones et al. 2000), endogenous proinflammatory proteins fibronectin (Bulut et al. 2002) HSP60 (Okamura et al. 2001), and synthetic lipopeptides of the hepatitis C virus (HCV)-core protein (Ohashi et al. 2000).

TLR4 is expressed in a wide range of tissues including liver, spleen, heart, lung, placenta, colon and small intestine (Zarember and Godowski 2002). Our data suggests that intrauterine starvation diminishes the postnatal TLR4 expression in the liver, which is a major target organ of LPS and other microbial products. Early observations demonstrated that liver is a primary site of uptake and clearance of microbial products, including LPS (reviewed in Duesberg et al. (2002)). Similar to macrophages, Kupffer cells are responsive to LPS, producing TNF-α and IL-1β that activate hepatocytes to express proinflammatory products such as the inducible nitric oxide synthase (Tanikawa et al. 1998). Hepatocytes express TLR4 (Vodovotz et al. 2001), CD14 (Kurose et al. 1996) and MD2 (Liu et al. 1998)

and can react to LPS directly. Recently, TLR4 has been shown to mediate the LPS-induced activation of cellular transcription factor, NF-κB, in hepatocytes (Vodovotz et al. 2001, Liu et al. 2002). Despite decreased TLR4 protein levels, LPS-induced TNF-α release was increased in the *ex vivo* hepatocytes obtained from the IUGR animals. These findings contradict the data reported in aged mice, and suggest that the IUGR effect on dysregulated LPS-responses may be due to an alteration in the intracellular signaling mechanisms rather than TLR4 alone. Alternatively, IUGR may suppress the inhibitory pathways to turn off the immune activation upon microbial antigen stimulation.

It is thought that an adverse intrauterine environment during a critical stage of development permanently alters or "programs" the development of fetal tissues, by influencing the gene expression, and this allows the fetus to survive but with adverse consequences in postnatal life. Interestingly, IUGR did not modulate the TLR4 expression in the spleen or intestine, which would most likely affect the survival of the fetus.

IUGR has been shown to cause prolonged defects in the adaptive immune responses. T cell numbers were reduced in IUGR infants and they remained low for at least 12 months (Chandra 1981). In later childhood the numbers of T lymphocytes were normal, but their proliferative capacity was significantly reduced and cutaneous hypersensitivity was minimal or absent (Ferguson 1978). Our data from the 60-d-old adult rats also suggests that IUGR has prolonged effects on TLR4 and proinflammatory cytokine expression, which cannot be corrected by adequate nutrient intake postnatally. Molecular mechanisms underlying the "prenatal programming of gene expression" are not clearly known; however, these observations may help explain the persistent abnormal adaptive immune responses in the IUGR population beyond the neonatal period.

One of the long-term consequences of intrauterine starvation is the increased risk of developing type 2 diabetes (Renshaw et al. 2002). It has recently been suggested that low-grade, chronic inflammation may play a role in the pathogenesis of insulin resistance in obesity and type 2 diabetes. This suggestion is based on the demonstration of elevated plasma concentrations of proinflammatory cytokines, such as interleukin-6 (IL-6) and TNF-α (Katsuki et al. 1998, Pickup and Crook 1998, Ravelli et al. 1998, Fernandez-Real et al. 2001, Mishima et al. 2001), as well as acute-phase reactants (McMillan 1989) in diabetes, increased expression of TNF-α mRNA in the adipose tissue (Hotamisligil et al. 1995) and skeletal muscle (Sagizadeh et al. 1996) of obese and diabetic patients, and protection from obesity-induced insulin resistance in mice lacking the TNF receptor function (Uysal et al. 1997). Raised plasma IL-6 concentrations, indicative of low-grade inflammation, have been suggested to represent the link between insulin resistance and vascular disease that characterize the metabolic syndrome (Pickup et al. 1997). Our data suggests that not only the baseline hepatic IL-1β and TNF-α mRNA expressions are higher in the adult IUGR offspring of intrauterine nutrient restricted rats but also their hepatic purified lipoprotein free LPS-induced TNF-α release is enhanced when compared to the controls. These data suggest that the calorie restricted IUGR may lead to the dysregulation of innate immune responses against microbial antigens, which may play a role in the development of adult onset type 2 diabetes mellitus and vascular disease.

Here we show that IUGR dysregulates the hepatic expression of TLR4, IL-1β and TNF-α expression as well as the LPS-induced TNF-α release, which may help explain the increased risk of sepsis in this cohort as well as the development of an insulin resistant phenotype. Starvation leading to calorie restriction is a common problem in the world, affecting not only the inhabitants of developing countries (CDC New Update, September 2002) but also in the Unites States approximately 11% of the households experience food insecurity and 3.3 million of these households (starvation 3.27%) report the presence of hunger (Mitka 2002). Understanding the effects of intrauterine starvation on innate immune system and TLRs may potentially help to develop novel therapeutic strategies to prevent and/or treat infections in this high-risk population. In addition, TLR ligands such as bacterial CpG DNA have been proposed as vaccine adjuvants (Jiang and Koganty 2003). Understanding the effect of IUGR on TLR expression and function may help to design more effective vaccines.

### Acknowledgements

The work cited here was supported by grants from the NIH, HD 41230 and HD 25024 (to SUD), and NIAID KO8AI51216, NICHD P30HD34610 and Howard Hughes Prime Award (to OE).

### References

Alves-Rosa F, Vulcano M, Beigie-Bompadre M, Fernandez G, Palermo M, Isturiz MA. 2002. Interleukin-1beta induces *in vivo* tolerance to lipopolysaccharide in mice. Clin Exp Immunol 128:221–228.

Barton GM, Medzhitov R. 2002. Control of adaptive immune responses by Toll-like receptors. Curr Opin Immunol 14:380–383.

Beach RS, Gershwin ME, Hurley LS. 1982. Gestational zinc deprivation in mice: Persistence of immunodeficiency for three generations. Science 218:469–471.

Beutler B. 2002. TLR4 as the mammalian endotoxin sensor. Curr Top Microbiol Immunol 270:109–120.

Beutler B, Du X, Poltorak A. 2001. Identification of Toll-like receptor 4 (Tlr4) as the sole conduit for LPS signal transduction: Genetic and evolutionary studies. J Endotoxin Res 7:277–280.

Biragyn A, Ruffini PA, Leifer CA, et al. 2002. Toll-like receptor 4-dependent activation of dendritic cells by beta-defensin 2. Science 298:1025–1029.

Bulut Y, Faure E, Thomas L, et al. 2002. Chlamydial heat shock protein 60 activates macrophages and endothelial cells through Toll-like receptor 4 and MD2 in a MyD88-dependent pathway. J Immunol 168:1435–1440.

Chandra RK. 1975a. Reduced secretory antibody response to live attenuated measles and poliovirus vaccines in malnourished children. Br Med J 2:583–585.

Chandra RK. 1975b. Fetal malnutrition and postnatal immunocompetence. Am J Dis Child 129:450–454.

Chandra RK. 1975c. Antibody formation in first and second-generation offspring of nutritionally deprived rats. Science 190:289–290.

Chandra RK. 1981. Serum thymic hormone activity and cell-mediated immunity in healthy neonates, preterm infants, and small-for-gestational age infants. Pediatrics 67:407–411.

Chandra RK. 2002. Nutrition and the immune system from birth to old age. Eur J Clin Nutr 56(Suppl 3):S73–S76.

Duesberg U, von dem Bussche A, Kirschning C, Miyake K, Sauerbruch T, Spengler U. 2002. Cell activation by synthetic lipopeptides of the hepatitis C virus (HCV)-core protein is mediated by Toll like receptors (TLRs) 2 and 4. Immunol Lett 84:89.

Ferguson AC. 1978. Prolonged impairment of cellular immunity in children with intrauterine growth retardation. J Pediatr 93:52–56.

Fernandez-Real JM, Vayreda M, Richart C, et al. 2001. Circulating interleukin-6 levels, blood pressure and insulin resistance in apparently healthy men and women. J Clin Endocrinol Metab 86:1154–1159.

Godfrey KM, Barker DJP, Osmond C. 1994. Disproportionate fetal growth and raised IgE concentration in adult life. Clin Exp Allergy 24:641–648.

Harju K, Glumoff V, Hallman M. 2001. Ontogeny of Toll-like receptors Tlr2 and Tlr4 in mice. Pediatr Res 49:81–83.

Hasselbalch H, Ersboll AK, Jeppesen DL, Nielsen MB. 1999. Thymus size in infants from birth until 24 months of age evaluated by ultrasound. A longitudinal prediction model for the thymic index. Acta Radiol 40:41–44.

Holemans K, Van Bree R, Verhaeghe J, Meurrens K, Van Assche FA. 1997. Maternal semistarvation and streptozotocin-diabetes in rats have different effects on the *in vivo* glucose uptake by peripheral tissues in their female adult offspring. J Nutr 127:1371–1376.

Hoshino K, Takeuchi O, Kawai T, et al. 1999. Cutting edge: Toll-like receptor 4 (TLR4)-deficient mice are hyporesponsive to lipopolysaccharide: evidence for TLR4 as the Lps gene product. J Immunol 162:374.

Hotamisligil GS, Arner P, Caro JF, Atkinson RL, Spiegelman BM. 1995. Increased adipose tissue expression of tumor necrosis factor-alpha in human obesity and insulin resistance. J Clin Investig 95:2409–2415.

Jiang ZH, Koganty RR. 2003. Synthetic vaccines: The role of adjuvants in immune targeting. Curr Med Chem 10:1423–1439.

Katsuki A, Sumida Y, Murashima S, et al. 1998. Serum levels of tumor necrosis factor-α are increased in obese patients with noninsulin-dependent diabetes mellitus. J Clin Endocrinol Metab 83:859–862.

Kawasaki K, Akashi S, Shimazu R, Yoshida T, Miyake K, Nishijima M. 2000. Mouse Toll-like receptor 4.MD-2 complex mediates lipopolysaccharide-mimetic signal transduction by Taxol. J Biol Chem 275:2251–2254.

Kurose I, Miura S, Higuchi H, et al. 1996. Increased nitric oxide synthase activity as a cause of mitochondrial dysfunction in rat hepatocytes: roles for tumor necrosis factor alpha. Hepatology 24:1185–1192.

Kurt-Jones EA, Popova L, Kwinn L, et al. 2000. Pattern recognition receptors TLR4 and CD14 mediate response to respiratory syncytial virus. Nat Immunol 1:398–401.

Liu S, Khemlani LS, Shapiro RA, Johnson ML, et al. 1998. Expression of CD14 by hepatocytes: Upregulation by cytokines during endotoxemia. Infect Immun 66:5089–5098.

Liu S, Gallo DJ, Green AM, et al. 2002. Role of Toll-like receptors in changes in gene expression and NF-kappa B activation in mouse hepatocytes stimulated with lipopolysaccharide. Infect Immun 70:3433–3442.

Matsumura T, Ito A, Takii T, Hayashi H, Onozaki K. 2000. Endotoxin and cytokine regulation of Toll-like receptor (TLR) 2 and TLR4 gene expression in murine liver and hepatocytes. J Interf Cytok Res 20:915–921.

McMillan DE. 1989. Increased levels of acute-phase serum proteins in diabetes. Metabolism 38:1042–1046.

Mishima Y, Kuyama A, Tada A, Takahashi K, Ishioka T, Kibata M. 2001. Relationship between serum tumor necrosis factor-α and insulin resistance in obese men with type 2 diabetes mellitus. Diabet Res Clin Pract 52:119–123.

Mitka M. 2002. Not enough food (instead of too much) is also a problem in the United States. JAMA 288:1462–1463.

Miyake K, Ogata H, Nagai Y, Akashi S, Kimoto M. 2000. Innate recognition of lipopolysaccharide by Toll-like receptor 4/MD-2 and RP105/MD-1. J Endotoxin Res 6:389–391.

Modlin RL. 2002a. Mammalian toll-like receptors. Ann Allergy Asthma Immunol 88:543–547.

Modlin RL. 2002b. Mammalian toll-like receptors. Ann Allergy Asthma Immunol 8:543–547.

Moore SE. 1998. Nutrition, immunity and the fetal and infant origins of disease hypothesis in developing countries. Proc Nutr Soc 57:241–247.

Moscatelli P, Bricarelli FD, Piccinini A, Tomatis C, Dufour MA. 1976. Defective immunocompetence in foetal undernutrition. Helv Paediatr Acta 31:241–247.

Muzio M, Bosisio D, Polentarutti N, et al. 2000. Differential expression and regulation of toll-like receptors (TLR) in human leukocytes. J Immunol 164:5998–6004.

Ohashi K, Burkart V, Flohe S, Kolb H. 2000. Cutting edge: Heat shock protein 60 is a putative endogenous ligand of the toll-like receptor-4 complex. J Immunol 164:558–561.

Okamura Y, Watari M, Jerud ES, et al. 2001. The extra domain A of fibronectin activates Toll-like receptor 4. J Biol Chem 276:10229–10233.

Phillips DIW, Cooper C, Fall C, et al. 1993. Fetal growth and autoimmune thyroid disease. Q J Med 86:247–253.

Pickup JC, Crook MA. 1998. Is type II diabetes mellitus a disease of the innate immune system? Diabetologia 41:1241–1248.

Pickup JC, Mattock MB, Chusney GD, Burt D. 1997. NIDDM as a disease of the innate immune system: Association of acute phase reactants and interleukin-6 with metabolic syndrome X. Diabetologia 40:1286–1292.

Poltorak A, He X, Smirnova I, et al. 1998. Defective LPS signaling in C3H/HeJ and C57BL/10ScCr mice: Mutations in Tlr4 gene. Science 282:2085.

Ravelli AC, van der Meulen JH, Michels RP, et al. 1998. Glucose tolerance in adults after prenatal exposure to famine. Lancet 351:173–177.

Renshaw M, Rockwell J, Engleman C, Gewirtz A, Katz J, Sambhara S. 2002. Cutting edge: Impaired toll-like receptor expression and function in aging. J Immunol 169:4697–4701.

Rietschel ET, Brade H, Holst O, et al. 1996. Bacterial endotoxin: Chemical constitution, biological recognition, host response, and immunological detoxification. Curr Top Microbiol Immunol 216:40–81.

Sagizadeh M, Ong JM, Garvey WT, Henry RR, Kern PA. 1996. The expression of TNF-alpha by human muscle: Relationship to insulin resistance. J Clin Investig 97:1111–1116.

Simchen MJ, Beiner ME, Strauss-Liviathan N, et al. 2000. Neonatal outcome in growth-restricted versus appropriately grown preterm infants. Am J Perinatol 17:187–192.

Tanikawa K, Mimura Y, Sakisaka S, Noguchi K. 1998. Role of hepatocytes in the clearance of lipopolysaccharide and its clinical significance. Prog Clin Biol Res 397:191–198.

Uysal KT, Wiesbrock SM, Marino MW, Hotamisligil GS. 1997. Protection from obesity-induced insulin resistance in mice lacking TNF-alpha function. Nature 389:610–614.

Vodovotz Y, Liu S, McCloskey C, Shapiro R, Green A, Billiar TR. 2001. The hepatocyte as a microbial product-responsive cell. J Endotoxin Res 7:365–7357.

Wenzel RP, Pinsky MR, Ulevitch RJ, Young L. 1996. Current understanding of sepsis. Clin Infect Dis 22:407–413.

Zarember KA, Godowski PJ. 2002. Tissue expression of human Toll-like receptors and differential regulation of Toll-like receptor mRNAs in leukocytes in response to microbes, their products, and cytokines. J Immunol 168:554–561.

